# Treatment of Dysentery

**Published:** 1853-05

**Authors:** 


*Treatment of Dysentery.*—Dr. J. B. Evans, of Frankfort, Ohio, in a communication for the North Western Med. and Surg. Journal, writes as follows:

After all is said, opium is the boon. I say *Opium.* It cannot be pressed too strongly. Sulphate of morphia seemed to be the best form. Without it the suffering of the patient was insupportable; he would die of actual pain. Providence is kind in giving us such a cordial for our sufferings. In many cases the amount of morphine used was very great. In my own case my attending friends gave me within a pittance of a dram in four weeks; the nervous irritability was so great that the strictest quiet, and the constantly soothing effect of opium was the only alternative. In these extremely nervous cases all treatment was useless, unless the strictest quiet was observed. Too many persons are allowed in the sick room. We should not let delicacy toward the friends of our patients interfere with our duty in this matter; for we are accountable for his life, so far as he is within the reach of means in our power.

*Nourishment.* The duration of an epidemic attack is usually beyond the starving point, and on this account, if no other, would arise the necessity for food. The starving plan of many of our professional brethren, I hope will be abandoned. Our diseases are becoming more grave and protracted, as the country becomes older and more densely inhabited. I have seen nothing more marked than the good effect derived from food in our scourge of dysentery. Nourishment was necessary from the commencement to the close of an attack. Even when the patient had no relish, and his feelings forbade him eating, he would be much better and suffer less after taking suitable nourishment. In many of the worst cases, the nervous system was completely unstrung, and having a constant griping, the horror produced is indescribable, except to the sufferer, who has felt it in his own person. The suffering was little short of a *fundamental* fire. In short, opium, quiet, and a judicious nourishment, were the great essentials in our epidemic. It was necessary to persist in a well-selected and oft-repeated nourishment, without which the fabric would fall, and I am satisfied that by its neglect many died.

